# Unveiling the Pharmacological Mechanisms of Eleutheroside E Against Postmenopausal Osteoporosis Through UPLC-Q/TOF-MS-Based Metabolomics

**DOI:** 10.3389/fphar.2020.01316

**Published:** 2020-08-26

**Authors:** Yong-Sheng Ma, Zhan-Jiang Hou, You Li, Beng-Beng Zheng, Jia-Ming Wang, Wen-Bo Wang

**Affiliations:** ^1^The Second Department of Orthopedics, The First Affiliated Hospital of Harbin Medical University, Harbin, China; ^2^The Emergency Surgery Department, The First Affiliated Hospital of Harbin Medical University, Harbin, China; ^3^The Third Department of Orthopedics, The First Affiliated Hospital of Harbin Medical University, Harbin, China

**Keywords:** metabolomics, ultra-high performance liquid tandem quadrupole time-of-flight mass spectrometry, osteoporosis, effects, pharmacological mechanism, biomarkers

## Abstract

Postmenopausal osteoporosis (PMOP) is a common metabolic bone disease in postmenopausal women in the Worldwide, and seriously affects the quality of life of middle-aged and elderly women. Therefore, there is an urgent need to discover a highly effective drug for PMOP treatment. In this study, ultra-high performance liquid tandem quadrupole time-of-flight mass spectrometry (UPLC-Q/TOF-MS) was used to analyze the urine metabolic profiling and potential biomarkers, the relevant metabolic network of PMOP rats, and further to evaluate the intervention effect of Eleutheroside E (EE) against PMOP. Using multivariate statistical analysis combined with UPLC-Q/TOF-MS, a total of 27 biomarkers were identified, which related with 16 metabolic pathways, mainly involving steroidogenesis, beta oxidation of very long chain fatty acids, glutathione metabolism, carnitine synthesis, estrone metabolism, oxidation of branched chain fatty acids, etc. After treatment of EE, these biomarkers were markedly regulated, mainly involving steroid hormone biosynthesis, arachidonic acid metabolism, primary bile acid biosynthesis, indicating that EE had the therapeutic effect on PMOP. This study identified the potential urine metabolic markers and related metabolic pathways of the PMOP, explained the metabolic effect and pharmacological mechanisms of EE against PMOP, and provided a basis for the pharmacological study of EE.

## Introduction

Osteoporosis (OP) is a systemic skeletal disease, which is characterized by a decrease in bone mass and destruction of bone tissue microstructure, leading to an increased risk of fracture (Eastell et al., 2016; Brown, 2017). OP is caused by the imbalance between osteoclast-induced bone resorption and osteoblast-induced bone formation (Dera et al., 2019; Yang et al., 2019), which is mostlyfound in middle-aged and elderly people, and there are nearly 200 million new patients every year (Yaacobi et al., 2017). After menopause, women’s estrogen deficiency leads to increased osteoclast activity, decreased bone density, and increased bone conversion, resulting in massive bone loss and ultimately postmenopausal osteoporosis (PMOP) (Mundy, 2001; Rachner et al., 2011). With the increasing aging population, the incidence of PMOP has also increased year by year, which seriously affects the quality of life of middle-aged and elderly people and greatly increases the burden of social medical care. Thus, the prevention and treatment of PMOP becomes particularly important. Currently, PMOP is mainly treated with estrogen-replacement therapy, bisphosphonates, calcium and vitamin D, calcitonin, selective estrogen receptor modulators or parathyroid hormone, but they often lead to different complications and adverse reactions in patients (Richard Eastell, 1998; Yoshiyuki et al., 2005). Therefore, a new drug with high efficiency and low toxicity for the prevention and treatment of PMOP is urgently needed.

A previous study showed that the Acanthophanax senticosus has efficacy on the ovariectomized osteoporosis model rats, could affect the endogenous metabolites related metabolic mechanism, and has provided a therapeutic basis for the PMOP treatment. Eleutheroside E (EE) is an important active ingredient from Acanthopanax senticosus, however, its therapeutic mechanism for PMOP is unclear. Metabolomics method was used to explore the potential biomarkers and therapeutic targets for natural products. Therefore, this study was to explore and evaluate the therapeutic targets of EE on the ovariectomized osteoporosis model rats by visually observe the dynamic changes of the urine metabolic profiling, and to find biomarkers and related metabolic pathways for PMOP, in order to provide experimental basis for the development of new anti-osteoporosis drugs.

## Experimental

### Materials and Reagents

Acetonitrile and methanol was purchased from Merck Drugs & Biotechnology (HPLC grade, Germany); formic acid was obtained from Kemiou Chemical Reagent Co. Ltd. (HPLC grade, Tianjin, China); ultrapure water was provided by Watson’s Food & Beverage Co., Ltd (Guangzhou, China). Nylestriol was provided from Vikchi Biotech Co., Ltd. (Sichuan, China); normal saline was provided by Harbin Medisan Pharmaceutical Co., Ltd. (Harbin, China). Chloral hydrate obtained from Guangfu Fine Chemical Research Institute (Tianjin, China). Sodium penicillin for injection was purchased from Harbin pharmaceutical Group Holding Co., Ltd. (Harbin, China). All other reagents are of analytical grade. The standard chemicals of EE with 99.5% was purchased from Chengdu Biopurity Phytochemicals Ltd., (Sichuan, China); and the chromatogram of EE was shown in **Figure S1**.

### Animals Handing

Wistar rats (female, weight 220–240g) were provided from the Experimental Animal Center of Harbin Medical University, and randomly divided into sham operation group (Sham, n=8), model group (M, n=8), the EE group (n=8), and positive control group (PC, n=8). After adapting to the environment, the rats were anesthetized by intraperitoneal injection of 10% chloral hydrate solution (0.3g·kg^-1^). Except for Sham, bilateral ovaries were removed by surgical emasculation in the remaining five groups to prepare PMOP rat model (Morvan et al., 2006). Sham rats were removed only the same weighting of fat around the ovaries. Rats in each group were deprived of food and water 12 h before surgery. After surgery, 5w IU penicillin sodium was injected intraperitoneally for 3 consecutive days. The postoperative convalescence period was 6 days, and the administration continued for 4 weeks from the 7th day. The EE rats were orally administrated-with EE of 0.1g·kg^-1^ every day. PC rats was oral administrated of nalestriol (1mg·kg^-1^) once a week, and distilled water in the remaining time. Sham and Model rats were given distilled water by gavage.

### Sample Collection

Urine in each group was collected for 12 h at night every 4 days for a total of 12 weeks. Fresh urine samples were initially processed by centrifugation (4°C, 10 min at 10,000 rpm), and the supernatant was frozen at -80°Cfor later using. Before analysis, urine samples were thawed at room temperature. After vortexing for 10 s, the samples were centrifuged at 10,000 rpm for 10 min at 4°C. The supernatant was passed through a 0.22 μm filter and transferred to a sample cup for UPLC-MS analysis.

### UPLC-MS Analysis

An ultra LC 100 system (AB. Ltd., USA) equipped with an ACQUITY UPLC™ T3 Column (100 mm×2.1 mm i.d., 1.8 μm, Waters Corp., USA) was used to analyze urine samples. The column temperature was maintained at 45°C. The 4 μl was set as the injection volume. And all samples had been kept at 4°C sample room during the data collection period. The mass spectrometry (MS) was performed usinga Triple TOF^R^ 5600^+^ high-definition quadrupole time-of-flight mass spectrometry (AB, USA) with electrospray ion (ESI) source. The full-scan mass number ranges from 50 to 1,000, and the eight strongest fragment ions over 100 CPS are scanned for the daughter ions. Dynamic background deduction was enabled, and the automatic correction system was automatically adjusted and corrected MS and MS/MS.

### Metabolomics Data Processing

The urine metabolic profiling data by UPLC-Q/TOF-MS was imported into Progenesis^R^ QI software (Waters, USA) and Metaboanalyst for peak alignment and standardization. The three-dimensional data of ion retention time– mass to charge ratio-peak intensity of metabolites were extracted. Metaboanalyst was used for multivariate statistical analysis. The biomarkers of PMOP were identified by searching various metabolomics databases such as Human Metabolome Database (HMDB), Kyoto Encyclopedia of Genes and Genomes (KEGG), and combining MS/MS fragmentation information. The identified compounds mass error range was ±5ppm. And then MetaboAnalyst 4.0 was performed for metabolic pathway analysis and visualization.

## Results

### Metabolic Profiling Analysis

**Figure 1:**
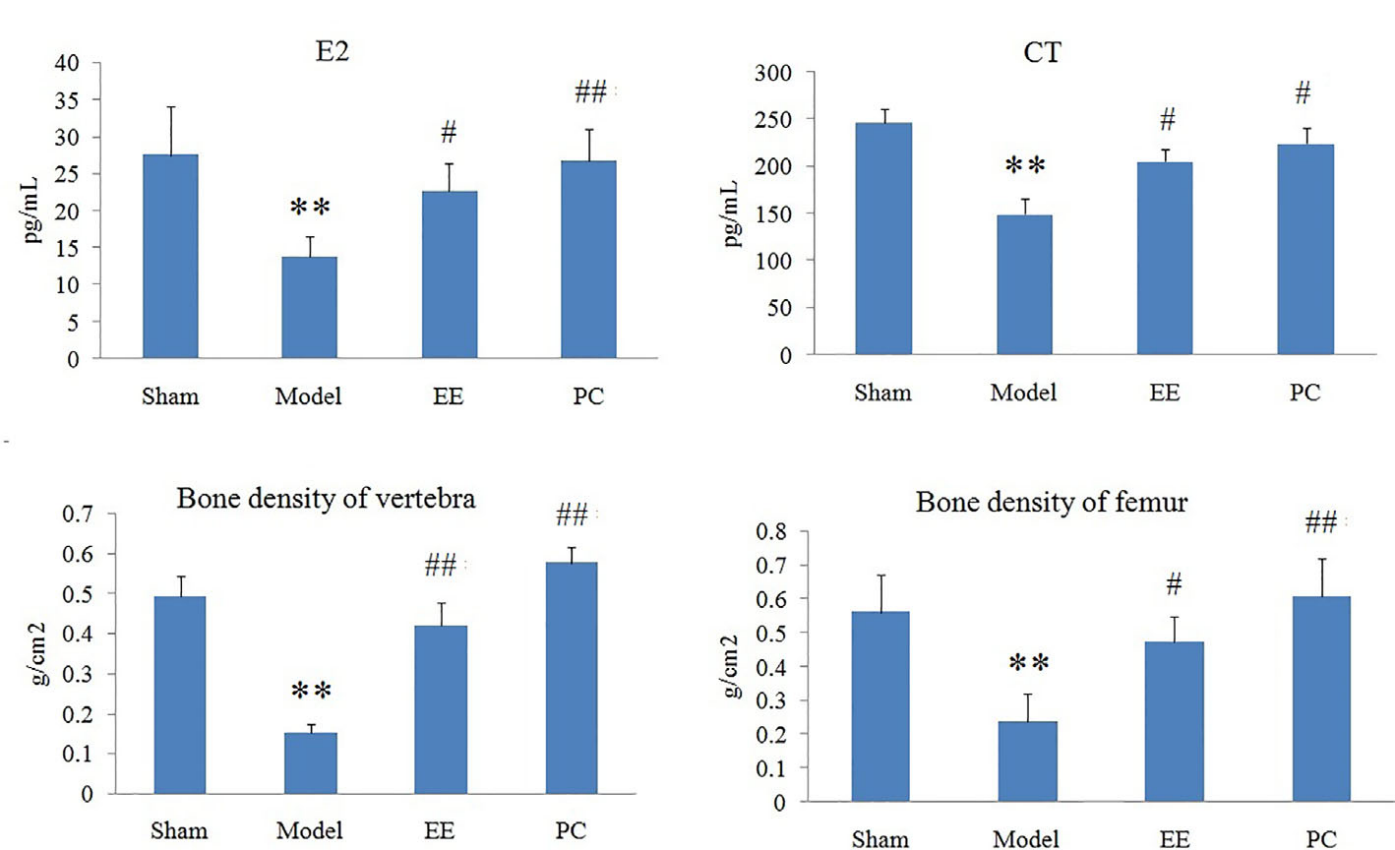
The principal component analysis (PCA) scores plot of urine samples from the control group and the model group. **P < 0.01 compared with sham group; ^##^*P* < 0.01, ^#^*P* < 0.05 compared with model group.

The previous studies displayed that PMOP rat model was replicated by the ovariectomy (Zhang et al., 2019). In this study, the urine metabolic profiling data of rats in M group on 28 days were imported into Progenesis^R^ QI software for data dimensionality reduction and MS matrix information acquisition. Metaboanalyst software was further used to carry out multivariate statistical analysis on the data of M group, and the principal component analysis (PCA) scores plot that could reflect the PMOP change process was obtained. Metaboanalyst software was used to analyze the urine metabolism profiling of M group and Sham group on the 28th day of model replication for PCA (**Figure 1**). It could be seen that M group and Sham group had obvious clustering and intergroup separation.

### Identification of PMOP Model Biomarkers

**Figure 2:**
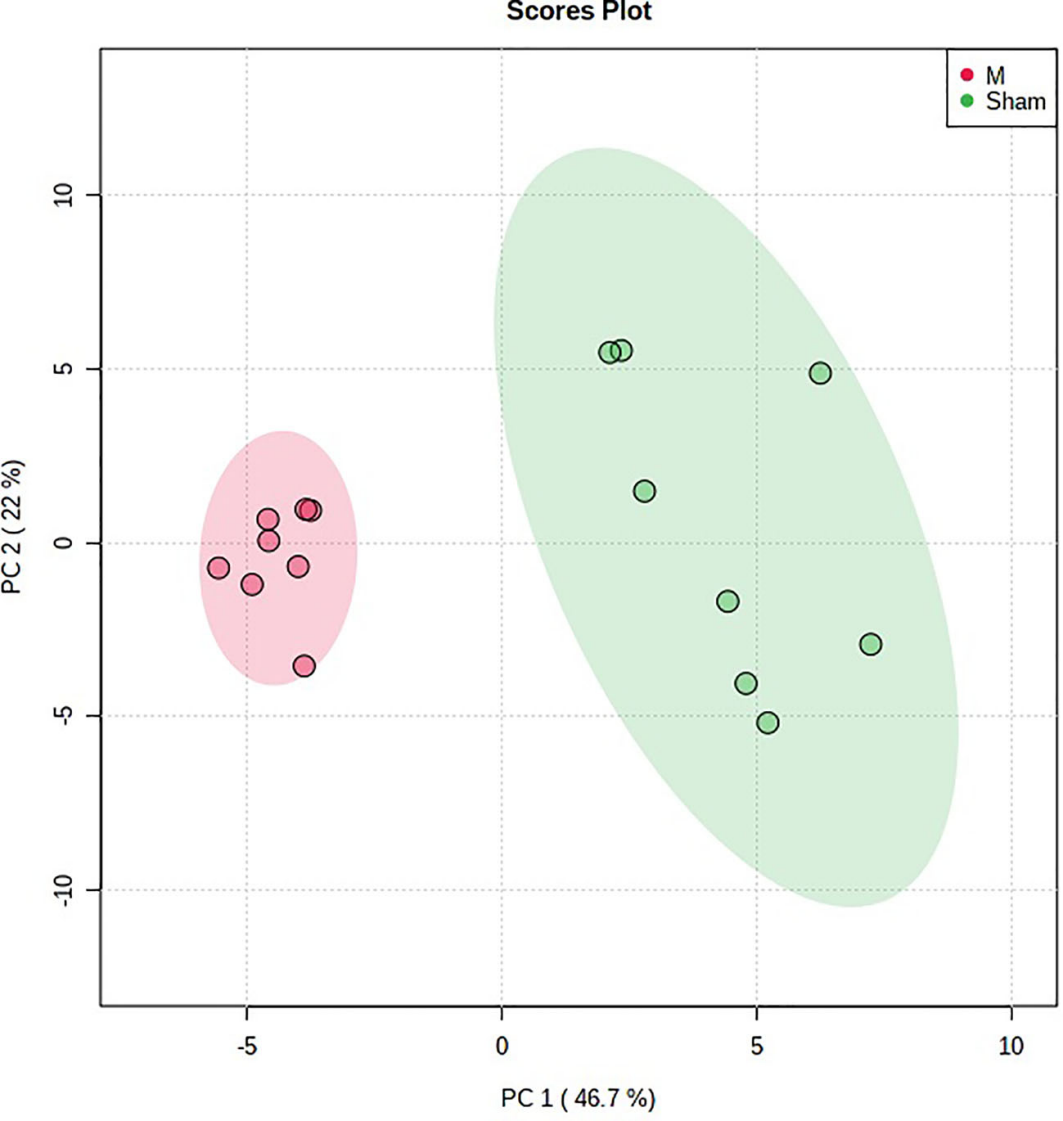
The heatmap analysis for urine potential biomarkers in the control group and the model group.

In order to search the potential endogenous metabolites that played the key role in clustering, orthogonal partial least squares discriminant analysis (OPLS-DA) was performed on the urine metabolic profiling data of rats in Sham and M groups to obtain the Scores plot. Ions with VIP-value greater than 1.0 and t-test results between groups (P-value) less than 0.05 were selected as potential biomarkers. Endogenous metabolites were screened by HMDB and KEGG databases, combined with MS/MS fragment information of compounds to determine the chemical structure of potential biomarkers. Finally, a total of 27 biomarkers were identified in PMOP model, including 15 in positive ion mode, 12 in negative ion mode. The details of identified biomarkers were shown in the **Table S1**. Furthermore, the statistical analysis module in the MetaboAnalyst database was used to perform clustering heat map analysis based on the relative abundance of the identified biomarkers to visualize the differences between Sham and M group (**Figure 2**).

### Metabolic Pathways Analysis

**Figure 3:**
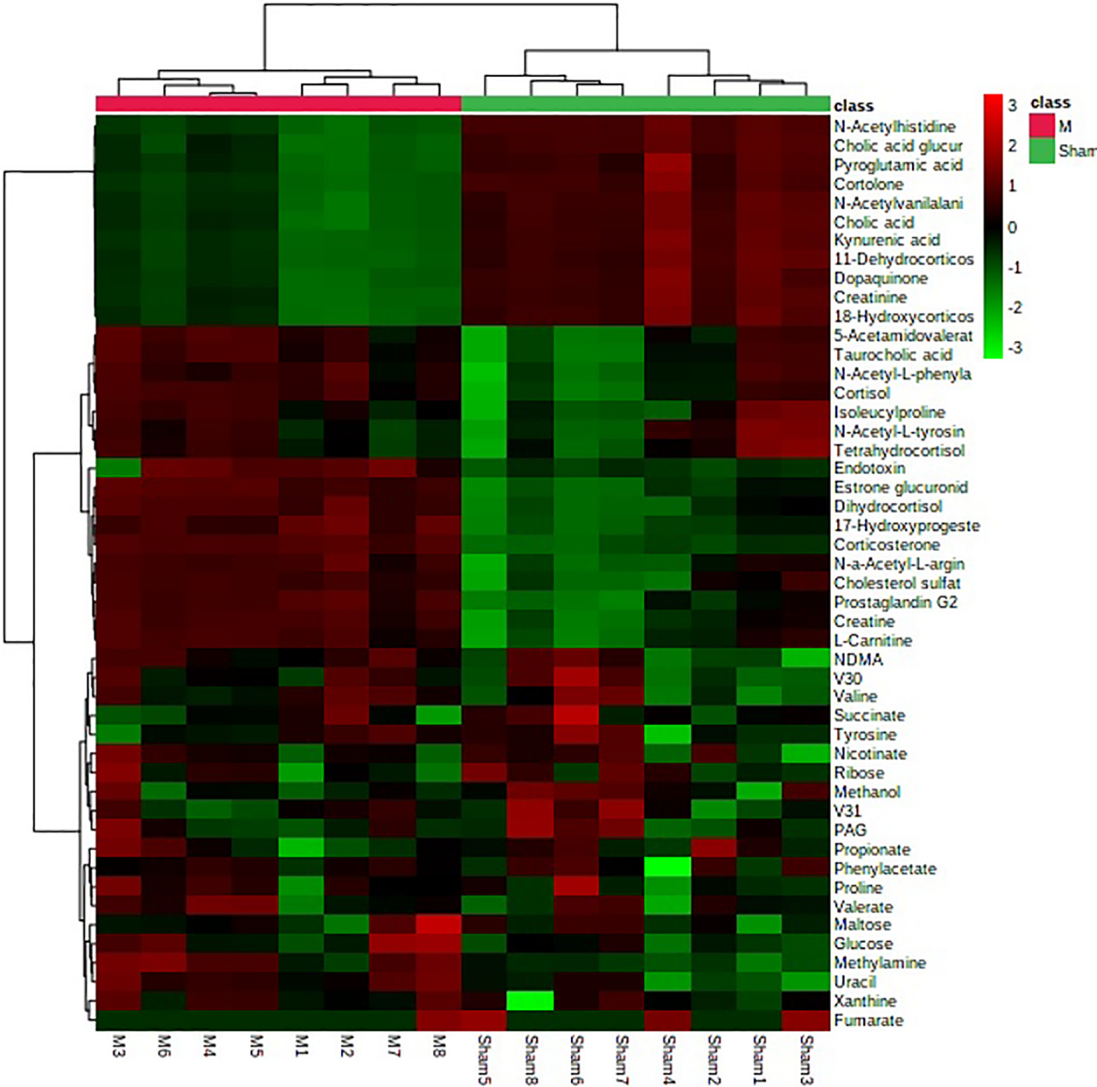
The metabolite sets enrichment overview of urine biomarkers in t postmenopausal osteoporosis.

**Figure 4:**
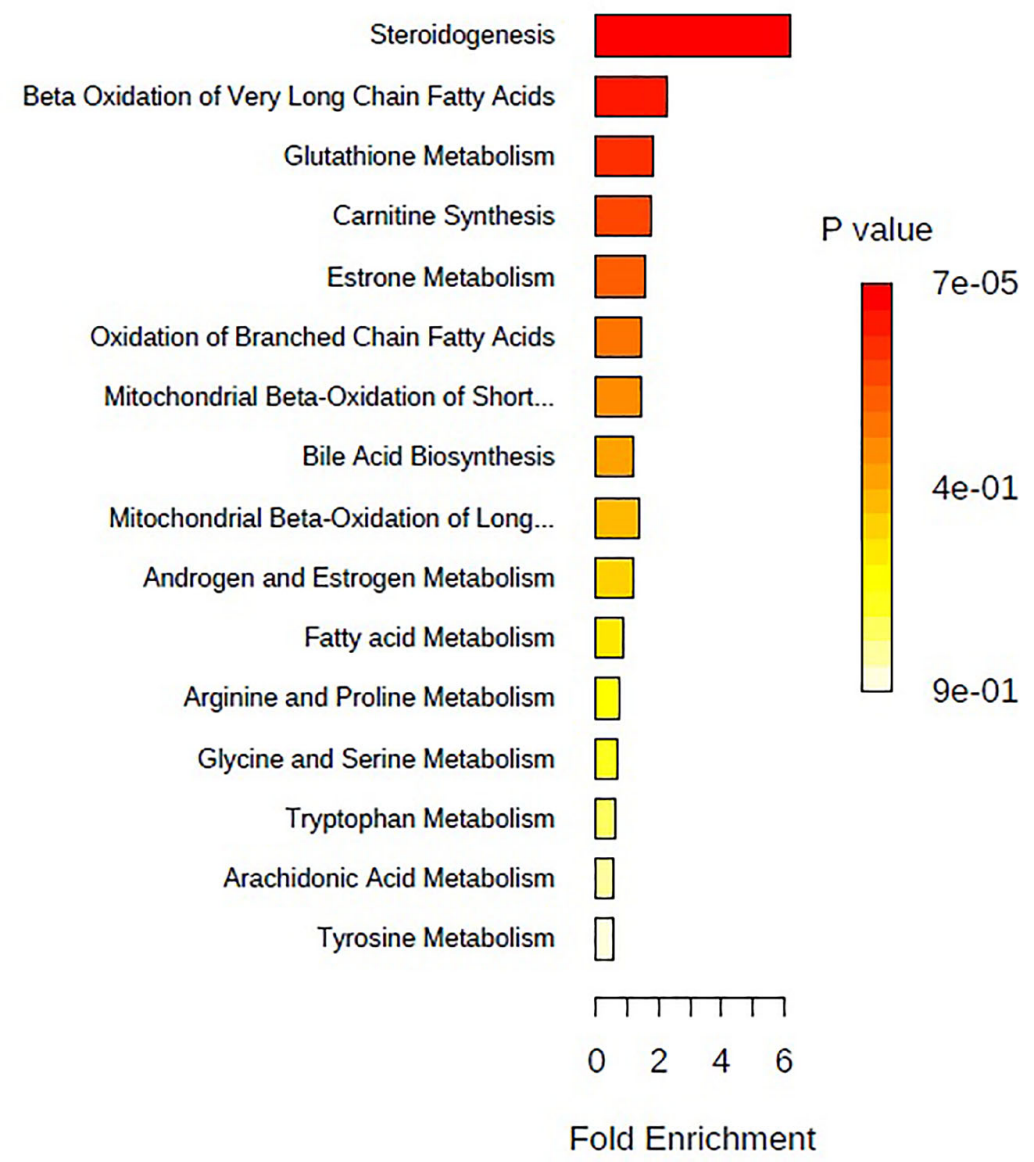
The network pathways analysis of urine biomarkers in postmenopausal osteoporosis by the MetaboAnalyst.

The above identified of 27 PMOP model biomarkers were imported into the Pathway Analysis module of MetaboAnalyst to analyze the metabolic network systematically. A total of 10 related metabolic pathways were acquired, including steroidogenesis, beta oxidation of very long chain fatty acids, glutathione metabolism, carnitine synthesis, estrone metabolism, oxidation of branched chain fatty acids, mitochondrial beta-oxidation of short chain saturated fatty acids, bile acid biosynthesis, mitochondrial beta-oxidation of long chain saturated fatty acids, androgen and estrogen metabolism, fatty acid metabolism, arginine and proline metabolism, glycine and serine metabolism, tryptophan metabolism, arachidonic acid metabolism, and tyrosine metabolism (**Figure 3**, **Table S2**). The metabolic network was drawn according to the correlation between disturbed biomarkers and metabolic pathways to observe visually the metabolic changes of PMOP (**Figure 4**).

### Therapeutic Effects of TL Against PMOP

**Figure 5:**
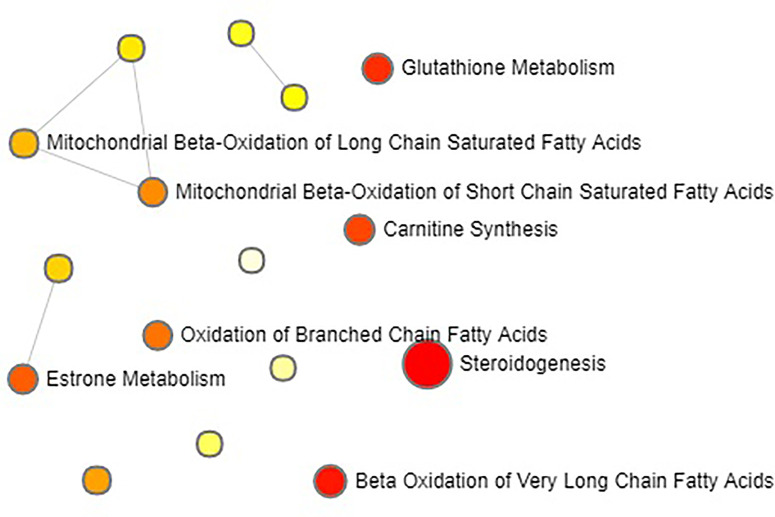
The principal component analysis (PCA) score plot of all the groups in urine metabolism profile by eleutheroside E against postmenopausal osteoporosis.

**Figure 6:**
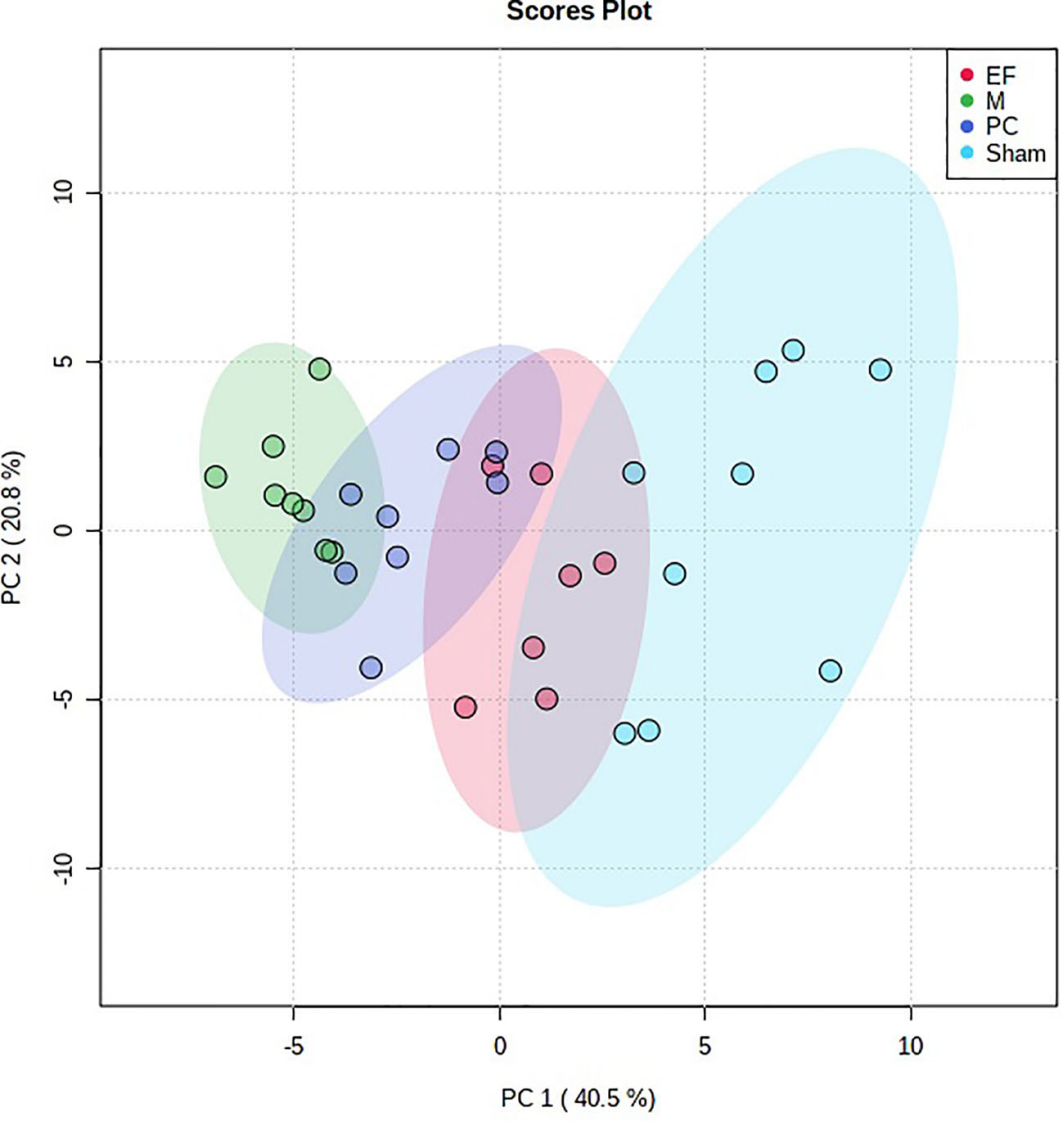
The principal component analysis (PCA) 3-D scores plot of urine metabolism profile samples from eleutheroside E against postmenopausal osteoporosis.

**Figure 7:**
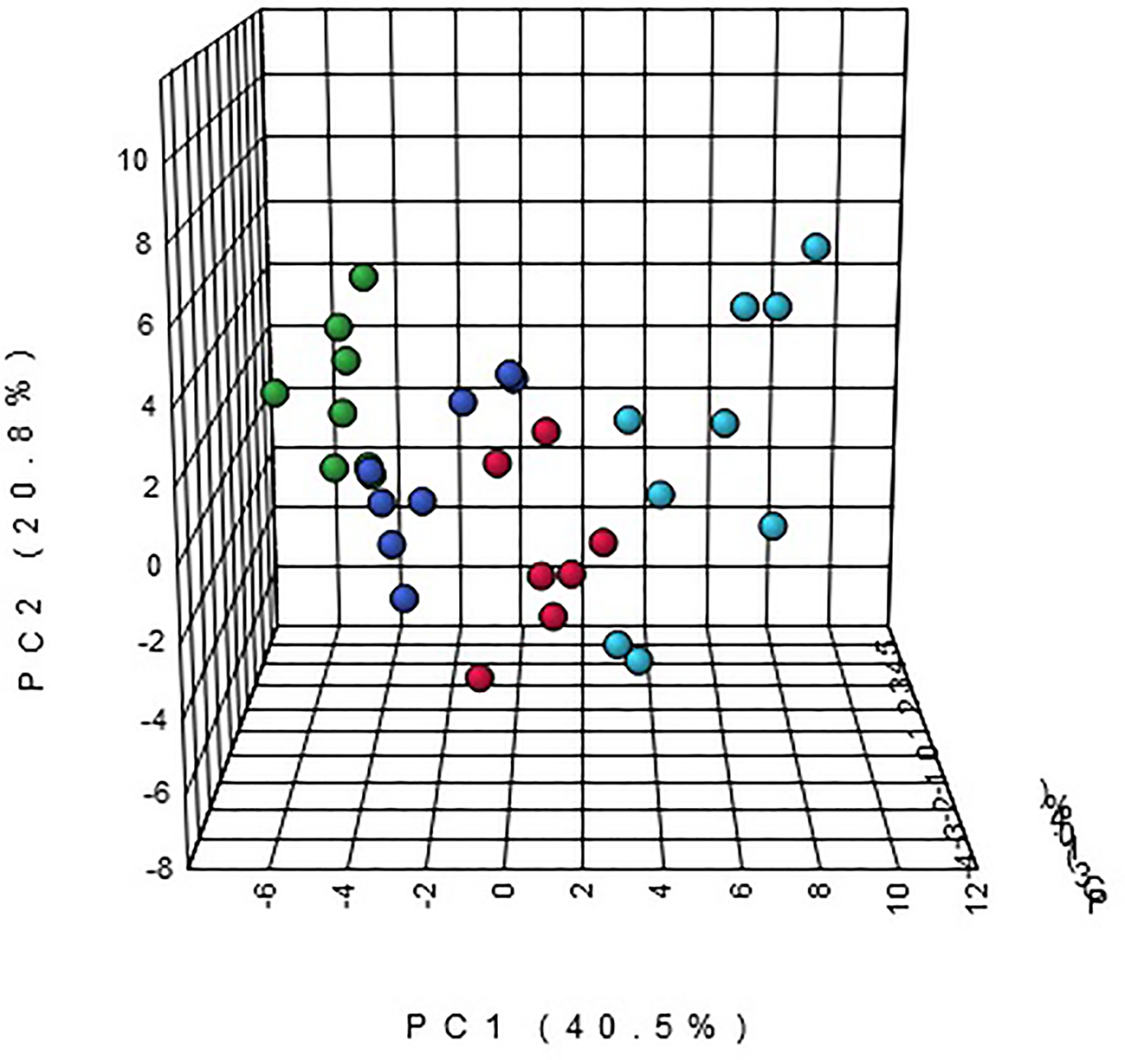
The heatmap analysis for urine potential biomarkers in eleutheroside E against postmenopausal osteoporosis.

**Figure 8:**
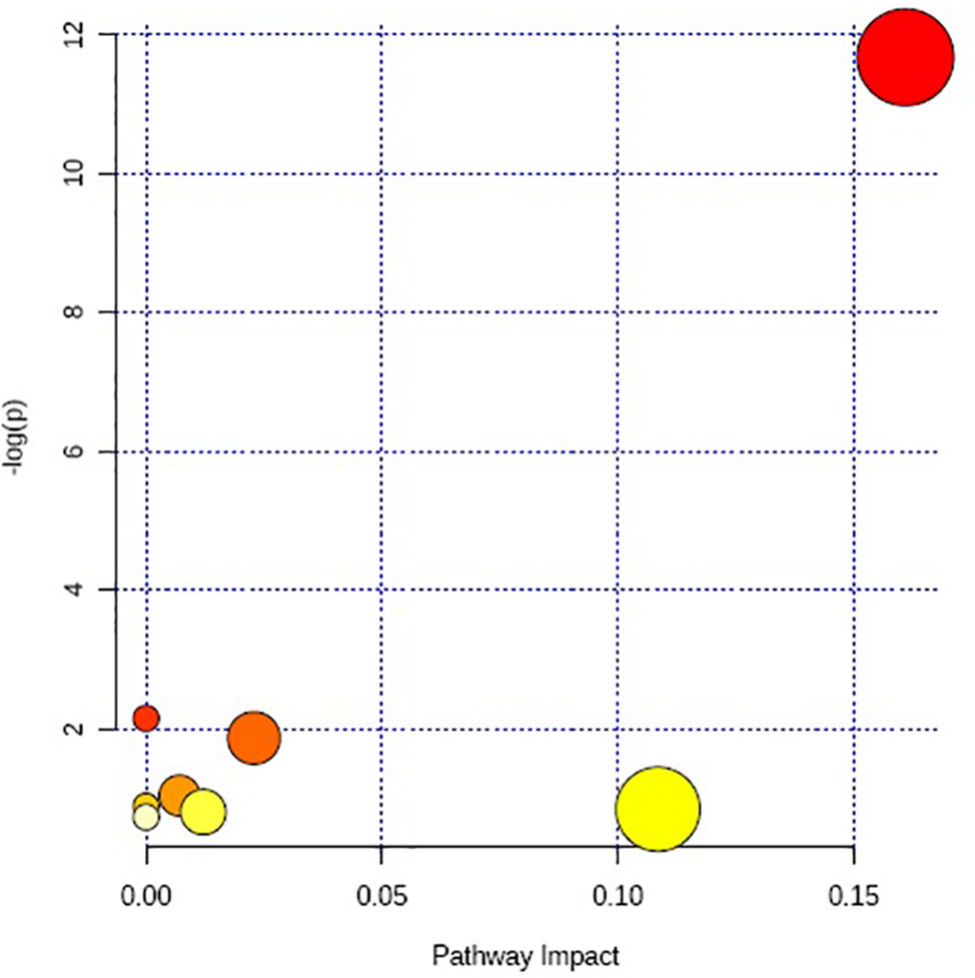
The metabolic pathways regulation analysis of eleutheroside E against postmenopausal osteoporosis.

Multivariate statistical analysis was performed on the urinary metabolic profiling data of rats in Sham, M, EE, and PC group on the 28th day. From the PCA scores plot (**Figure 5**), it can be seen that the samples in each group showed clustering in the group and separation between groups, sham group was remarkably separated from M group, and metabolic profiling of the rats in each group changed significantly after treatment (**Figure 6**). Among that, the vector position of PC and TL-M groups was far away from M group, and was closest to Sham group. It could be seen from the changes in the content of metabolites that the EE can regulate 21 biomarkers, and nilestriol can regulate 22 biomarkers (**Figure 7**). After treatment of EE, these biomarkers were markedly regulated, mainly involving steroid hormone biosynthesis, arachidonic acid metabolism, primary bile acid biosynthesis (**Figure 8**, **Table S3**). These results indicated that EE could effectively regulate the pathological state of PMOP, and the pharmacological effects was similar to nilestriol treatment.

## Discussions

In this study, we established a UPLC-Q/TOF-MS metabolomics method to analyze urine samples of PMOP model rats, and observed the endogenous metabolites changes in the urine of rat models. From the perspective of metabolomics, the normal metabolic network of rats was disturbed after model replication, and the metabolic process in PMOP rat model was also demonstrated. It can be seen that the metabolic profiling of the model rats had a large change on the 28th day after model replication. Then the established analysis method was used to analyze the urine metabolic profiling of M group and Sham group on the 28th day of model replication. The two groups were clearly separated, which indicates that the PMOP model was successfully replicated from the metabolomics level. Next, using multivariate statistical analysis combined with multiple metabolomics databases and matching MS/MS fragmentation information, 27 potential biomarkers related to PMOP were found, resulting in disturbances of 10 metabolic pathways, mainly involving lipid metabolism and amino acid metabolism.

The disturbance of lipid metabolism was dominant, includingsteroid hormone biosynthesis, arachidonic acid metabolism, and primary bile acid biosynthesis. Research suggests that there are many factors play a role in the formation of the PMOP, including metabolic disorders, especially hormonal imbalances (Al-Anazi et al., 2011). In this study, metabolomics method was used to confirm that PMOP formation is accompanied by significant hormone imbalance, resulting in significant changes in the level of 21 metabolites in the steroid hormone biosynthesis, which seriously affects the steroid hormone biosynthesis pathway. This pathway is mainly involved in hormone synthesis in the body, including estrogen, androgens, glucocorticoids, and mineralocorticoids. Estrogen is produced by the ovaries and placenta of female animals. The ovary, asgonad of the female, has reproductive and endocrine functions, can secrete a variety of hormones and growth factors to control the nine major systemsof the body which bones, immunity, reproductionand so on. Its normal functioning is affected by the cerebral cortex, hypothalamus, and pituitary (Elie et al., 2020). After ovariectomy, the level of estrogen in the body decreases rapidly, and the levels of other hormones secreted by the ovaries also show significantdrops. At the same time, the hypothalamic-pituitary-ovarian (HPO) axis system is destroyed, and the hypothalamus and pituitary cannot be regulated by positive feedback, leading to the synthesis or secretion of hypothalamus gonadotropin-releasing hormone (GnRH) and pituitary gonadotropin are reduced, which in turn affects the synthesis and secretion of sex hormones in the body. In addition to its reproductive effects, estradiol plays an important role in regulating bone growth, maturation, and bone turnover (Hajime et al., 1995; Miyauchi et al., 2013). Lack of estradiolwill leads to increased osteoclast formation, enhanced bone resorption, and loss of cancellous and cortical bone, resultingto the occurrence of PMOP (Brennan et al., 2014). Natural estrogens are mainly estradiol, estrone, and estriol, which can beconverted into each other *in vivo*. The level of estradiol in the female body is the highest at reproductive age, estriol during pregnancy, and estrone during menopause. Estradiol and estrone are easily combined with glucuronic acid, which increases water solubility and easy to be excreted by the urine. It was found that the content of estroneglucuronide in the urine of ovariectomized rats was significantly reduced in the study, indicating that the estrogen level in ovariectomized rats decreased, which was consistent with the changes of hormone level in postmenopausal women.Adrenocortical hormones are a class of hormones produced by the adrenal cortex stimulated by adrenocorticotropic hormones secreted from the anterior pituitary gland, including glucocorticoids and mineralocorticoids. Cortisol and corticosterone are adrenocortical hormones produced by the adrenal cortex, which are a type of glucocorticoid. Their synthesis and secretion are affected by the hypothalamic-pituitary-adrenal (HPA) axis (Kamani et al., 2017). Studies have shown that the HPA axis activity disorders playthe key role in the pathogenesis of age-related OP (Reynolds et al., 2005). After ovariectomy, the level of estrogen decreases, resulting in abnormal function of HPA axis, and reduced adrenal function, furtherdecreases the secretion of glucocorticoids by the adrenals. Metabolomics studies confirmed that the secretion of cortisol, corticosterone, and their metabolites in the urine of ovariectomized rats was significantly reduced.

Prostaglandin G2 (PGG2) is the main product synthesized from arachidonic acid by cyclooxygenase (COX) and can be further metabolized to prostaglandin E2 (PGE2) (Simmons et al., 2004). Prostaglandin E (PGE) is an important factormediating osteoblast bone synthesis signaling pathway, which plays a wide role in bone formation, bone healing and inflammatory bone loss in human body. PGE can promote bone formation by stimulating osteoblasts, thus improving bone quality. PGE2 is a recognized substance that promotes the proliferation of osteoblasts. It can activate the intracellular cAMP-mediated pathway by coupling with the G protein receptor subtype, and then activate the downstream molecules of the signaling pathway to participate in the formation of new bone (Mirsaidi et al., 2017); and it can promote bone absorption by down-regulating the gene expression of bone protective proteinof osteoblasts (Chen et al., 2019). The results showed that the secretion of PPG2 in urine of ovariectomized OP rats was significantly reduced, which may lead to a decrease in the production of PGE2, which affected bone resorption and bone formation.

Cholesterol is an indispensable and important substance for animal tissue cells, and is the raw material for the synthesis of bile acids and steroid hormones. It can bind to estrogen receptor-associated receptor alpha topromote osteoclast formation, survival, and cell fusion (Zheng et al., 2020). Cholic acid and chenodeoxycholic acid are synthesized bycholesterol in the liver, and then combines with glycine or taurine to form the conjugated primary bile acids. Bile acids are mainly present in the hepato-intestinal circulatory system and re-absorbed into the liver for reuse, and play an important role in fat and fat-soluble vitamin metabolism (Boyer, 2013). Studies have shown that PMOP is associated with turnover rate ofbile acid (Hanly et al., 2013). And fat-soluble vitamin A and vitamin D are also related to OP. Vitamin A can inhibit osteoblast activity and activate osteoclast activity (Kindmark et al., 1995; Saeed et al., 2019). Vitamin D is an important bone metabolism regulating hormone, which is involved in bone deposition, bone growth, bone reconstruction, and can improve bone density (Lorentzon et al., 2001; Kruger et al., 2012). In addition, after the ovaries are removed in rats, the cholesterol synthesis hormone pathway is affected, resulting in abnormal bile acid synthesis and excretion (Bouchard et al., 1993). From the results of metabolomics studies, it can be seen that the secretion of cholesterol sulfate, cholic acid, cholic acid glucuronide, and taurocholic acid in urine is significantly increased, indicating that cholesterol metabolism is affected by ovarian deficiency, causing abnormal bile acid conversion, which directly affected primary bile acid biosynthesis, indirectly affected the absorption of vitamin A and D, leading to bone loss and OP.

The formation of PMOP is accompanied by significant disturbances in lipid metabolism, as well as significant disturbances in amino acid metabolism. Amino acid metabolism plays an important role in bone metabolism (Zhao et al., 2018). Tyrosine is one of the amino acids that make up proteins, which is hydroxylated by phenylalanine *in vivo*. The phosphorylation of protein tyrosine is controlled by the synergistic action of protein tyrosine phosphatases (PTPs) and protein tyrosine kinases. PTPs are an important factor involved in osteoclast and osteoblast activities in bone metabolism (Schmidt et al., 1996; Shalev and Elson, 2019). Dopaquinone is produced by the oxidation of dopa and is one of the tyrosine metabolites, which is involved in various metabolic disorders. Homogentisic acid is an intermediate in the decomposition or catabolism of tyrosine and phenylalanine. High levels of homogentisic acid can produce osteotoxin and nephrotoxin, which is a substance that causes bone and joint damage (Kraus, 2015). Homogentisic acid can be oxidized to generate gentisate aldehyde, which is a substrate of aldehyde oxidase 1 in the pathways of valine, leucine and isoleucine degradation, tyrosine metabolism, and nicotinic acid and nicotinamide metabolism. The levels of dopaquinone, homogentisic acid, and gentisate aldehyde in the urine of ovariectomized rats were significantly increased, affecting the bone metabolism, resulting in bone damage and osteoporosis.

Arginine, proline, leucine, and phenylalanine can enhance the effect of insulin-like growth factor 1 (Liu et al., 2008; Yang et al., 2010) (IGF-1), which can promote the proliferation and differentiation of osteoblasts (Zhang et al., 2012; Reckenbeil et al., 2017). Creatine is mainly synthesized by arginine, glycine and methionine in the liver. Creatinine is the final product of creatine and phosphocreatine metabolism. The excretion of creatinine reflects the function of the kidney. In addition, studies have shown that creatinine is associated with OP (Eisenhauer et al., 2019).The production of creatinine is constant, and the elimination of creatinine and creatinine in the urine was reduced, this result indicates that the kidney is impaired and the excretion of creatinine is blocked. Abnormal renal function can cause endocrine system disorders, affecting the function of osteoblasts and osteoclasts.

Pyroglutamic acid is produced byglutathione (GSH) under the action of CHAC, it can produce *L*-glutamic acid bypyroglutaminase, and *L*-glutamic acid is also producedby GSH under the action of glutathione hydrolase. The decrease of estrogen secretion and the increase of reactive oxygen species (ROS) are important reasons for the occurrence of PMOP (Hamidi et al., 2012; Cervellati et al., 2014). When OP occurs, cells are in astate of oxidative stress, and the damaged cells are increases, leading todecrease the activity of osteoblasts and enhance the activity of osteoclasts, and bone reconstruction is blocked. GSH has anti-oxidant properties, which can remove free radicals, reduce cell damage, and promote repair and reconstruction of bone cells (Haruo et al., 2004). The content of pyroglutamic acid and *N*-acetylglutamine in urine decreased significantly, indicating that GSH and *L*-glutamic acid of its upstream substances are affected, the body was in an oxidative stress state, estrogen secretion were reduced, and ROS were increased and weakened antioxidant capacity lead to increased cell damage, unbalanced bone reconstruction, decreased bone density, and damaged bone microstructure, resulting in OP.

Ovariectomized rats were administrated of EE for 28 consecutive days, 21 metabolites were call backed, among which 11 metabolites were significantly regulated, namely pyroglutamic acid, N2,N2-dimethylguanosine, dopaquinone, 5-acetamidovalerate, taurocholic acid, cholesterol sulfate, 17-hydroxyprogesterone, cholic acid, cholic acid glucuronide, 7a,12a-dihydroxy-3-oxo-4-cholenoic acid, 21-deoxycortisol. It mainly involves steroid hormone biosynthesis, primary bile acid biosynthesis, glutathione metabolism, and tyrosine metabolism. These results show that EE might inhibit cholesterol to synthesize bile acid, enhance the selective binding ability of EE to estrogen receptor, and promote the function of adrenaline, and then alleviate the dysfunction of HPA axis and increase the secretion of glucocorticoid; at the same time, EE might also reduce cell damage by adjusting the oxidative stress state in the body. Under the action of insulin and EGF regulatory factors, the bone density and bone strength of the ovariectomized rats were increased, and bone reconstruction was promoted, thus delaying the PMOP process. Combined with the previous results of blood metabolomics (Shalev and Elson, 2019), it is suggested that the primary bile acid biosynthesis may be a targeted regulatory pathway of EE.

## Conclusions

In this study, with the multivariate statistical analysis and UPLC-Q/TOF-MS, a total of 27 biomarkers were identified, which related with 16 metabolic pathways, mainly involving steroidogenesis, beta oxidation of very long chain fatty acids, glutathione metabolism, carnitine synthesis, estrone metabolism, oxidation of branched chain fatty acids, etc. These biomarkers were markedly regulated by EE, mainly involving steroid hormone biosynthesis, arachidonic acid metabolism, primary bile acid biosynthesis, indicating that EE had the therapeutic effect on PMOP. This study identified the potential urine metabolic markers and related metabolic pathways of the PMOP, explained the metabolic effect and pharmacological mechanisms of EE against PMOP, and provided a basis for the pharmacological study of EE.

## Data Availability Statement

The raw data supporting the conclusions of this article will be made available by the authors, without undue reservation, to any qualified researcher.

## Ethics Statement

The animal study was reviewed and approved by First Affiliated Hospital of Harbin Medical University.

## Author Contributions

W-BW conceived and designed the experiments. Y-SM, Z-JH, YL, B-BZ, and J-MW performed the experiment and analyzed the data. Y-SM wrote the paper. All authors contributed to the article and approved the submitted version.

## Conflict of Interest

The authors declare that the research was conducted in the absence of any commercial or financial relationships that could be construed as a potential conflict of interest.
