# Supplementary material for: Unveiling the Pharmacological Mechanisms of Eleutheroside E Against Postmenopausal Osteoporosis Through UPLC-Q/TOF-MS-Based Metabolomics
Source: Front Pharmacol. 2020 Aug 26;11:1316. doi: 10.3389/fphar.2020.01316 (PMC7479840; doi:10.3389/fphar.2020.01316)
Supplement: Supplementary file 1 [file DataSheet_1.docx]

**Table S1** Detailed information of the identified biomarkers in positive and negative ion modes.

| **NO.** | **Rt.** | **m/z** | **Mass error (ppm)** | **Compounds** | **Element composition** | **Ion form** |
| --- | --- | --- | --- | --- | --- | --- |
|  |  |  |  |  |  |  |
| 1 | 0.89 | 132.0764 | 0.9 | Creatine | C_4_H_9_N_3_O_2_ | [M+H]^+^ |
| 2 | 0.97 | 162.1127 | 1.1 | *L-*Carnitine | C_7_H_15_NO_3_ | [M+H]^+^ |
| 3 | 1.27 | 112.0665 | 0.5 | Creatinine | C_4_H_7_N_3_O | [M-H]^-^ |
| 4 | 1.35 | 198.0871 | 1.2 | N-Acetylhistidine | C_8_H_11_N_3_O_3_ | [M+H]^+^ |
| 5 | 1.62 | 130.0494 | 2.0 | Pyroglutamic acid | C_5_H_7_NO_3_ | [M+H]^+^ |
| 6 | 1.65 | 215.1298 | 1.4 | *N*-a-Acetyl-*L*-arginine | C_8_H_16_N_4_O_3_ | [M-H]^-^ |
| 7 | 1.86 | 229.1541 | 0.5 | Isoleucylproline | C_11_H_20_N_2_O_3_ | [M+H]^+^ |
| 8 | 2.65 | 254.1026 | 0.4 | *N*-Acetylvanilalanine | C_12_H_15_NO_5_ | [M+H]^+^ |
| 9 | 3.40 | 194.0463 | 1.2 | Dopaquinone | C_9_H_9_NO_4_ | [M-H]^-^ |
| 10 | 4.22 | 160.0829 | 2.2 | 5-Acetamidovalerate | C_7_H_13_NO_3_ | [M+H] ^+^ |
| 11 | 4.25 | 222.0778 | 0.5 | *N*-Acetyl-*L*-tyrosine | C_11_H_13_NO_4_ | [M-H]^-^ |
| 12 | 4.29 | 190.0497 | -0.6 | Kynurenic acid | C_10_H_7_NO_3_ | [M+H]^+^ |
| 13 | 5.77 | 367.2473 | -1.3 | Cortolone | C_21_H_34_O_5_ | [M+H]^+^ |
| 14 | 6.07 | 445.1898 | 0.7 | Estrone glucuronide | C_24_H_30_O_8_ | [M-H]^-^ |
| 15 | 6.13 | 206.0828 | 0.7 | *N*-Acetyl-*L*-phenylalanine | C_11_H_13_NO_3_ | [M-H]^-^ |
| 16 | 6.27 | 363.2163 | -0.6 | Cortisol | C_21_H_30_O_5_ | [M+H]^+^ |
| 17 | 6.59 | 363.2317 | -0.9 | Dihydrocortisol | C_21_H_32_O_5_ | [M-H]^-^ |
| 18 | 6.46 | 363.2162 | -0.5 | 18-Hydroxycorticosterone | C_21_H_30_O_5_ | [M+H]^+^ |
| 19 | 7.35 | 514.2833 | -0.3 | Taurocholic acid | C_26_H_45_NO_7_S | [M-H]^-^ |
| 20 | 7.48 | 465.3046 | -0.5 | Cholesterol sulfate | C_27_H_46_O_4_S | [M-H]^-^ |
| 21 | 7.60 | 345.2054 | -0.5 | 11-Dehydrocorticosterone | C_21_H_28_O_4_ | [M+H]^+^ |
| 22 | 7.84 | 331.2257 | -0.4 | 17-Hydroxyprogesterone | C_21_H_30_O_3_ | [M+H]^+^ |
| 23 | 7.98 | 365.2325 | -0.6 | Tetrahydrocortisol | C_21_H_34_O_5_ | [M-H]^-^ |
| 24 | 8.09 | 409.2789 | -1.4 | Cholic acid | C_24_H_40_O_5_ | [M+H] + |
| 25 | 8.27 | 583.3115 | -1.0 | Cholic acid glucuronide | C_30_H_48_O_11_ | [M-H]^-^ |
| 26 | 8.34 | 347.2216 | -1.4 | Corticosterone | C_21_H_30_O_4_ | [M+H]^+^ |
| 27 | 8.63 | 367.2119 | -0.2 | Prostaglandin G2 | C_20_H_32_O_6_ | [M-H]^-^ |

**Table S2** Metabolite sets enrichment analysis of urine biomarkers in postmenopausal osteoporosis.

| Name | total | expected | hits | Raw p |
| --- | --- | --- | --- | --- |
| Steroidogenesis | 43 | 1.13 | 7 | 0.00 |
| Beta Oxidation of Very Long Chain Fatty Acids | 17 | 0.45 | 1 | 0.37 |
| Glutathione Metabolism | 21 | 0.55 | 1 | 0.43 |
| Carnitine Synthesis | 22 | 0.58 | 1 | 0.45 |
| Estrone Metabolism | 24 | 0.63 | 1 | 0.48 |
| Oxidation of Branched Chain Fatty Acids | 26 | 0.69 | 1 | 0.51 |
| Mitochondrial Beta-Oxidation of Short Chain Saturated Fatty Acids | 27 | 0.71 | 1 | 0.52 |
| Bile Acid Biosynthesis | 65 | 1.71 | 2 | 0.52 |
| Mitochondrial Beta-Oxidation of Long Chain Saturated Fatty Acids | 28 | 0.74 | 1 | 0.53 |
| Androgen and Estrogen Metabolism | 33 | 0.87 | 1 | 0.59 |
| Fatty acid Metabolism | 43 | 1.13 | 1 | 0.69 |
| Arginine and Proline Metabolism | 53 | 1.40 | 1 | 0.77 |
| Glycine and Serine Metabolism | 59 | 1.56 | 1 | 0.80 |
| Tryptophan Metabolism | 60 | 1.58 | 1 | 0.81 |
| Arachidonic Acid Metabolism | 69 | 1.82 | 1 | 0.85 |
| Tyrosine Metabolism | 72 | 1.90 | 1 | 0.86 |

**Table S3** The metabolic pathways regulation analysis of eleutheroside E against postmenopausal osteoporosis.

| Name | Total | Expected | Hits | Raw p | Impact |
| --- | --- | --- | --- | --- | --- |
| Steroid hormone biosynthesis | 77 | 1.18 | 8 | 0.00 | 0.16 |
| Arachidonic acid metabolism | 36 | 0.55 | 1 | 0.43 | 0.11 |
| Primary bile acid biosynthesis | 46 | 0.70 | 2 | 0.15 | 0.02 |
| Arginine and proline metabolism | 38 | 0.58 | 1 | 0.45 | 0.01 |
| Glutathione metabolism | 28 | 0.43 | 1 | 0.35 | 0.01 |
| Taurine and hypotaurine metabolism | 8 | 0.12 | 1 | 0.12 | 0 |
| Glycine, serine and threonine metabolism | 34 | 0.52 | 1 | 0.41 | 0 |
| Tyrosine metabolism | 42 | 0.64 | 1 | 0.48 | 0 |


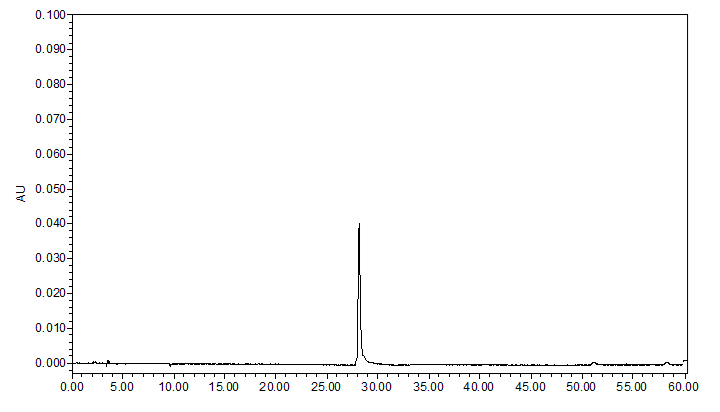


**Fig.S1.** The chromatogram of EE standard solution. Agilent 1200 high performance liquid chromatography with Agilent 1200 series pump. Chromatographic column: agilent Zorbax SB-C18 (250mm X 4.6 mm, 5um); detection wavelength: 220nm; mobile phase: 0.5% phosphoric acid aqueous solution-methanol (0-10 min, 9:91; 10-60 min, 9-25:91-75); column temperature: 25℃; the flow rate: 1.0 mL/min-1, the injection volume: 10uL.
